# MicroRNA profiling of dogs with transitional cell carcinoma of the bladder using blood and urine samples

**DOI:** 10.1186/s12917-017-1259-1

**Published:** 2017-11-15

**Authors:** Michael S. Kent, Allison Zwingenberger, Jodi L. Westropp, Laura E. Barrett, Blythe P. Durbin-Johnson, Paramita Ghosh, Ruth L. Vinall

**Affiliations:** 1 Department of Surgical and Radiological Sciences, School of Veterinary Medicine, University of California, Davis, CA USA; 2 Department of Medicine and Epidemiology, School of Veterinary Medicine, University of California, Davis, CA USA; 3 William R. Pritchard Veterinary Medical Teaching Hospital, School of Veterinary Medicine, University of California, Davis, CA USA; 4 Department of Public Health Sciences, University of California Davis, Davis, California, 95616 USA; 5 Department of Urology, University of California, Davis, School of Medicine, Sacramento, CA USA; 6 Department of Biochemistry and Molecular Medicine, University of California, Davis, School of Medicine, Sacramento, CA USA; 7 VA Northern California Health Care System, Sacramento, CA USA; 8 Department of Pharmaceutical and Biomedical Sciences, California Northstate University College of Pharmacy, Elk Grove, CA USA

**Keywords:** microRNA, Canine bladder cancer, Urine and blood analysis

## Abstract

**Background:**

Early signs of canine transitional cell carcinoma (TCC) are frequently assumed to be caused by other lower urinary tract diseases (LUTD) such as urinary tract infections, resulting in late diagnosis of TCC which could be fatal. The development of a non-invasive clinical test for TCC could dramatically reduce mortality. To determine whether microRNAs (miRNAs) can be used as non-invasive diagnostic biomarkers, we assessed miRNA expression in blood and/or urine from dogs with clinically normal bladders (*n* = 28), LUTD (*n* = 25), and TCC (*n* = 17). Expression levels of 5 miRNA associated with TCC pathophysiology (miR-34a, let-7c, miR-16, miR-103b, and miR-106b) were assessed by quantitative real-time PCR.

**Results:**

Statistical analyses using ranked ANOVA identified significant differences in miR-103b and miR-16 levels between urine samples from LUTD and TCC patients (miR-103b, *p* = 0.002; and miR-16, *p* = 0.016). No statistically significant differences in miRNA levels were observed between blood samples from LUTD versus TCC patients. Expression levels of miR-34a trended with miR-16, let-7c, and miR-103b levels in individual normal urine samples, however, this coordination was completely lost in TCC urine samples. In contrast, co-ordination of miR-34a, miR-16, let-7c, and miR-103b expression levels was maintained in blood samples from TCC patients.

**Conclusions:**

Our combined data indicate a potential role for miR-103b and miR-16 as diagnostic urine biomarkers for TCC, and that further investigation of miR-103b and miR-16 in the dysregulation of coordinated miRNA expression in bladder carcinogenesis is warranted.

**Electronic supplementary material:**

The online version of this article (10.1186/s12917-017-1259-1) contains supplementary material, which is available to authorized users.

## Background

Transitional cell carcinoma (TCC) is the most common bladder tumor in dogs representing approximately 2% of all canine tumors [1–3]. Further, the prevalence of this disease is increasing [4, 5]. The recognized causes of TCC are varied with identified risk factors including use of topical insecticides, living in houses where the yards have been treated with insecticides, and living near marshes sprayed for mosquitoes or industrial areas [6, 7]. Female dogs and obese dogs appear to have an increased incidence of disease, in contrast to humans where the disease is more prevalent among male patients [3, 7]. There are also several breed predilections for TCC, including Scottish terriers, beagles, Shetland sheep dogs, wire fox terriers and West Highland white terriers, suggesting a genetic component to this disease [4, 8].

The extent of tumor locally as well as locoregional and distant metastasis is staged using the Tumor, Lymph Node and Metastasis (TNM) staging system [9]. Most dogs are diagnosed with TCC later in the course of disease with higher stage tumors being most common [10]. It has also been shown that having a higher stage of disease can negatively affect response to treatment. Treatment for TCC involves the use of surgery, radiotherapy, nonsteroidal anti-inflammatory agents and chemotherapy. Even with aggressive therapy, most dogs fail treatment and die from their disease [3, 11–17]. Some of the biggest gains in survival in human cancer medicine have occurred because of early detection of disease. This has proven true for prostate cancer, breast cancer and colon cancer as well as TCC. While ~80% of human patients present with superficial TCC, 10–30% of these patients will progress to invasive TCC and frequent screening has allowed for earlier detection of progression and has helped improve outcomes including survival [18–20]. Given this, early detection of TCC in dogs is likely to impact their response and survival.

Currently the diagnosis of TCC is most commonly made after dogs show advanced signs of disease, including hematuria, stranguria, and pollakiuria, all of which can mimic lower urinary tract disease (LUTD) [1]. Abdominal ultrasound may reveal structural changes to the urinary bladder, but lacks specificity and is also relatively expensive [4, 21]. Aspirates and open surgical biopsy of bladder tumors carry the risk of distant seeding of tumor cells [22, 23]. Other methods of definitive diagnosis, including traumatic catheterization and cystoscopically obtained biopsies, carry their own risks of complication and associated costs, making their use as a screening tool impractical [21]. The V-BTA rapid latex agglutination urine dipstick test to detect dogs with TCC proved not to be useful due to a low positive predictive value, resulting in only 3% of positive tests occurring in dogs with TCC [4, 24]. This indicates the need for an improved diagnostic screening test to detect dogs with TCC vs LUTD.

MicroRNAs (miRNAs) are small, highly stable, non-coding RNAs which facilitate post-transcriptional control of gene expression [25]. Multiple studies have demonstrated that dysregulation of miRNA expression levels can play a functional role in the initiation and progression of many cancers, and that miRNA can be used as diagnostic, prognostic, and predictive biomarkers [26]. Importantly, miRNA are relatively stable in most body fluids meaning they have the potential to be used clinically as non-invasive biomarkers [27–29]. The majority of miRNA biomarker discovery studies have focused on assessing miRNA levels in blood, and several blood-based miRNA biomarkers are currently in development. Assessment of miRNA levels in urine is possible but has proved much more challenging due to accelerated RNA degradation as a result of RNA being in an acidic environment [30–32]. As urine is in direct contact with the bladder (and bladder cancers, if present), urine samples are more likely than blood samples to contain miRNA which are derived from bladder cells and therefore any alterations in miRNA expression which are observed in urine samples are likely to reflect actual changes in miRNA expression which are occurring in bladder cells.

Altered expression of several miRNA has been observed in human bladder cancer progression [33–37]. We previously determined that assessment of miRNA in canine tissue specimens can be used to distinguish between inflammatory disease and TCC [38]; a statistically significant difference in expression levels of miR-34a, miR-16, miR-103b and miR-106b was observed between tissue specimens from canine LUTD and TCC patients. All of these miRNA have the ability to control expression of molecules which play a role in driving human bladder cancer initiation and progression, including components of the p53, Rb and/or Bcl-2 signaling pathways and likely play a role in driving bladder carcinogenesis in canines [39–45]. In addition, we recently demonstrated that the miRNA let-7c plays an important role in the response of human patients with bladder cancer to chemotherapy [46]. Based on these results, the goal of the current study was to determine whether assessment of these same miRNA is feasible in canine blood and urine samples, and whether these miRNA show potential for use as non-invasive biomarkers which can distinguish between non-neoplastic LUTD, such as urinary tract infections (UTI) and cystic calculi, and TCC, in canine patients.

The development of a non-invasive method versus an invasive method to distinguish between common inflammatory LUTD and TCC in canine patients is highly desirable for both economical and feasibility reasons and could have implications for human medicine [30–32, 47, 48].

## Methods

### Patient samples

Dogs with newly diagnosed, untreated transitional cell carcinoma of the urinary bladder and two control groups, clinically normal dogs and dogs with non-neoplastic lower urinary tract disease (such as dogs with UTI and cystic calculi), were entered into the study.

### Definitions of disease

A clinically normal dog was defined as a dog with no lower urinary tract signs, a normal bladder on ultrasound examination done by a board certified veterinary radiologist, a normal urinalysis and a negative urine culture result. Dogs were only enrolled if they were not receiving any antibiotics, non-steroidal or steroidal medications. Dogs with inflammatory or infectious lower urinary tract disease were defined as dogs with a positive urine culture and/or urolithiasis and no bladder mass seen under ultrasound evaluation, during surgical exploration of the bladder or during cystoscopy. Dogs with TCC were defined as dogs having either a histopathological or cytological diagnosis of transitional cell carcinoma. Only dogs not receiving any treatment for this disease including chemotherapy, non-steroidal anti-inflammatory medications or antibiotics at the time of sampling could be enrolled. As part of the study design, the person performing the molecular analyses was blinded as to the group inclusion of the sample until after sample analyses were complete.

### Analysis of canine blood

2.5 mls of whole blood was obtained from the jugular vein of each dog and placed in a PAXgene RNA Blood collection tube (Qiagen - Cat# 762165). PAXgene tubes were then inverted 8–10 times and stored at room temperature (20C) for 2 h before being transferred to -20C for 24 h. After 24 h, the tubes were transferred to -80C and stored until the time of analysis. The PAXgene blood miRNA kit (PreAnalytiX – Cat# 762165) was used to isolate RNA from blood samples per manufacturer’s instructions. To allow for normalization during subsequent qPCR analysis, a synthetic RNA was added to RNA preps (5.6 × 10^8^ copies of cel-miR-39 (Qiagen, Cat# 217184) per sample). This is a well accepted method for normalization [49].

### Analysis of canine urine

10mls of urine was collected by ultrasound guided antepubic cystocentesis. 3 mls was submitted for urinalysis, 2 mls was used for urine culture, and 5 mls was stored in 1 ml aliquots at −80 °C until the time of analysis. Prior to RNA extraction, urine was thawed on ice then centrifuged at 250xG for 5 min to pellet exfoliated cells present in the urine. The Qiagen miRNeasy kit (Qiagen – Cat# 217004) was used to extract RNA from cell pellets.

### miRNA analysis

RNA extracted from blood and urine extractions was quantified using a NanoDrop 2000 spectrophotometer. Relative expression levels of miR-34a, let-7c, miR-16, miR-103b, and miR-106b were assessed using predesigned TaqMan primer/probes sets (Applied Biosystems) in combination with the TaqMan MicroRNA Reverse Transcription and Universal PCR Master Mix (no AmpErase UNG) kit (Applied Biosystems, Cat# 4324018) per manufacturer’s protocol. Three replicates were included for each sample. Twenty nanograms of total RNA was used for each RT reaction. Cel-miR-39 expression levels were assessed to allow for normalization of miRNA expression in blood samples. To allow for normalization of miRNA expression in urine samples, RNU6 expression levels were assessed using predesigned TaqMan primer/probes sets (Applied Biosystems)). RNU6 is frequently used as an endogenous control gene for miRNA expression studies [50]. Expression values of miR-34a, let-7c, miR-16, miR-103b, and miR-106b are expressed relative to these normalization controls using the 2(−delta delta cycle threshold) method.

### Statistical analysis

We determined that inclusion of 20 normal, 20 LUTD, and 17 TCC patients would provide a power of 0.8 for our study (28 normal, 25 LUTD, and 17 TCC patients were included in the actual study). Data from each of the groups were graphed and parametric and/or nonparametric analyses performed to generate descriptive and inferential statistical data using a commercially available software program (GraphPad Prism, GraphPad Software, La Jolla, CA). The following variables were assessed in this study; age (continuous variable), weight (continuous variable), miRNA levels (miR-34a, let-7c, miR-16, miR-103b, miR-106b, continuous variable), gender (categorical variable (male/female)), presence of cystic calculi (categorical variable (yes/no)), presence of UTI (categorical variable (yes/no)). The Chi-squared test was used to determine whether differences in patient characteristics existed between groups for gender, while standard ANOVA was used to determine whether differences in age, weight, and amount of miRNA isolated from patient blood and urine specimens existed between groups. MiRNA expression levels were compared between groups using Kruskal-Wallis One Way ANOVA on Ranks (Ranked ANOVA), a method which is becoming common place in biomarker discovery studies because it is able to take into account the different levels of variation which are present in the groups being assessed [51–56]. It is of note that this type of analysis is considered exploratory in nature, i.e. is used for hypothesis-generation. Correlation between miRNA expression in the 3 patient groups was estimated by Pearson Product moments analysis. Statistical significance for all tests was set at *p* < 0.05.

## Results

### Patient characteristics

A total of 70 dogs, including 28 normal control dogs, 25 dogs with LUTD and 17 dogs with TCC were included in the study (Table 1). A statistically significant difference in mean age was observed between the groups (Table 1, *P* < 0.0001). Statistically significant differences in gender and weight were not observed (Table 1). In the normal control group there were 11 male castrated dogs and 17 female spayed dogs. In the LUTD control group there were 11 male castrated dogs, 1 intact male dog, 10 female spayed dogs and 3 intact female dogs. In the TCC group there were 9 male castrated dogs and 8 female spayed dogs. In the normal control group there were 15 mixed breed dogs, 3 Labrador retrievers, 2 border collies, 2 dachshunds and 1 each of 6 different pure bred dogs. All had negative urine cultures results and all had no evidence of LUTD on abdominal ultrasound. In the LUTD group there were 5 mixed breed dogs, 3 Bishon Frises, 2 pit bull terriers and 1 each of 14 different pure bred dogs. For the dogs with LUTD, 11 were diagnosed with cystic calculi, 11 were diagnosed with both cystic calculi and a urinary tract infection based on a positive aerobic bacterial urine culture, and 2 were diagnosed with chronic urinary tract infections. In the TCC group there were 4 mixed breed dogs, 2 Australian shepherds, 2 German shepherd dogs, 2 West Highland white terriers and 1 each of 7 different pure bred dogs. Five were diagnosed on biopsy and 12 were diagnosed on cytology. All dogs were assumed to have muscle invasive disease based on appearance on ultrasound examination but as not all dogs had biopsies taken this could not be fully evaluated. Three had a concurrent urinary tract infection based on results of aerobic bacterial urine cultures.

**Table 1:** Patient Characteristics

# Patients	Normal control	Dogs with LUTD	Dogs with TCC	*p*-value
	28	25	17	
Gender				
Male	11 (39.3%)	12 (48%)	9 (53%)	>0.5
Female	17 (60.7%)	13 (52%)	8 (47%)	>0.5
Characteristics				
Mean Age (Range) years	5.2 ± 2.98 (0.67–12.0)	7.5 ± 3.9 (0.5–14.0)	10.0 ± 2.4 (6–14)	<0.0001
Mean Weight (Range) Kg	17.85 ± 10.9 (3.4–40.0)	14.15 ± 12.16 (2.9–50.0)	20.97 ± 15.37 (5.1–69.0)	>0.5
Disease				
Cystic Calculi (CC)	0	11	0	
Urinary Tract infection (UTI)	0	2	3	
CC + UTI	0	11	0	
TCC based on biopsy	0	0	5	
TCC based on cytology	0	0	12	

### Extraction of miRNA from canine urine and blood samples

Based on recent publications comparing the levels of miRNA in blood and urine of bladder cancer patients [57–59], we assessed both blood and urine samples. Bladder cells are in direct contact with urine and as a number of the cells present in urine are of urothelial origin; the higher content of bladder cells in urine versus blood makes it more likely that observed changes in miRNA expression in urine samples are reflective of alterations that directly mediate bladder inflammation or TCC.

**Fig. 1:**
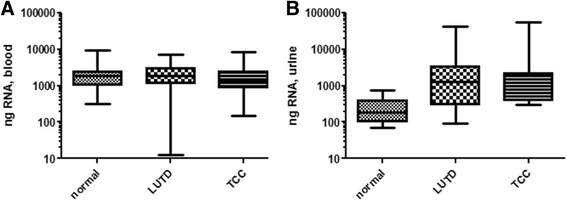
Relative quantities of RNA extracted from blood and urine specimens from canine patients with normal bladder, lower urinary tract disease (LUTD), and transitional cell carcinoma (TCC) of the bladder. Statistically significant differences in the quantity of RNA isolated from blood specimens (**a**) or urine specimens (**b**) from the 3 patient groups were not observed. However, there was a statistically significant difference in the quantity of RNA isolated from blood versus urine from patients with normal bladders (*p* = 0.0001). No statistically significant difference was observed in quantity of RNA isolated from blood versus urine from patients with LUTD, or patients with TCC. Standard ANOVA was used for these comparisons

We chose to isolate RNA from cells present in urine samples rather than isolate RNA which is present ‘free’ in the urine because other studies have demonstrated isolating sufficient amounts of high quality ‘free’ RNA from urine is challenging due to low pH and high levels of nucleases [30–32]. The median quantities of RNA isolated from cells present in canine patient urine samples from the three groups were: 181.6 ng (Normal patients, range; 70.8 ng – 760.4 ng, *n* = 28), 938.6 ng (LUTD patients, range; 93.6 ng – 42,337.6, *n* = 20), 1192.4 ng (TCC patients, range; 117.6 ng – 55,468.8 ng, *n* = 11) (Fig. 1A). There was not a statistically significant difference in the quantity of RNA isolated from the 3 patient groups.

The Qiagen PAXGene kit was used to isolate RNA from canine blood samples. The median quantities of RNA isolated from canine patient blood samples from the three groups were: 1839.8 ng (Normal patients, range; 320 ng – 9510.8 ng, n = 28), 1999.2 ng (LUTD patients, range; 12.4 ng – 7279.6 ng, *n* = 25), 1887.2 ng (TCC patients, range; 146.4 ng – 8636.6 ng, *n* = 17) (Fig. 1B). There was not a statistically significant difference in the quantity of RNA isolated from the 3 groups.

A statistically significant difference in RNA yields from blood versus urine samples collected from normal patients was observed (*p* = 0.0001, 10-fold difference), however, there was not a statistically significant difference in RNA yield for LUTD or TCC patients (Fig. 1A, B).

### Age-related differences in miR-34a expression in normal dogs and those with LUTD but not TCC

**Fig. 2:**
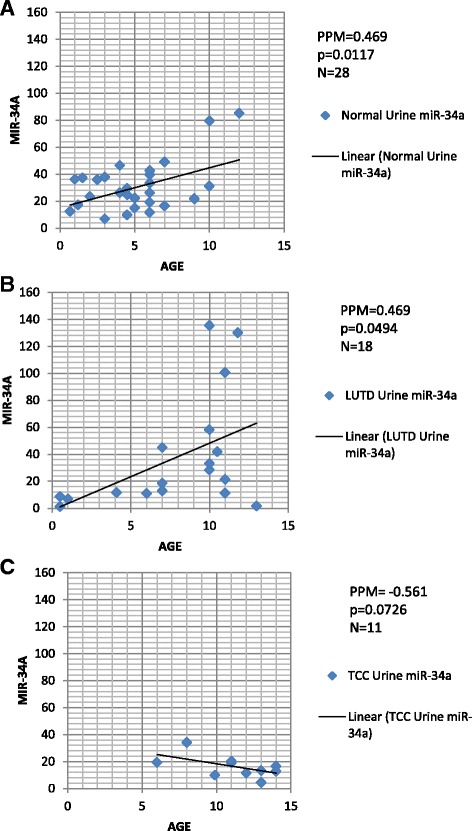
Age-related differences in miR-34a expression exist in urine samples from normal dogs and those with lower urinary tract disease (LUTD) but not in urine samples from dogs with transitional cell carcinoma (TCC) of the bladder. Urine miR-34a expression correlated strongly with age in both the normal group (**a**) and the LUTD group (**b**), but not in the TCC group (**c**). There were no age-related differences observed in let-7c, miR-16, miR-103b, or miR-106b expression (data not shown). A correlation between age and miRNA expression was not observed in canine blood samples (data not shown). Pearson Product Moment Correlation was used to generate these data

Expression of 5 miRNAs associated with TCC pathophysiology (miR-34a, let-7c, miR-16, miR-103b, and miR-106b) [38, 60], was assessed in RNA extracted from clinically normal, LUTD, and TCC canine blood and urine samples using quantitative real time PCR. Since a statistically significant difference in age was observed between dogs with normal bladder, LUTD and TCC (Table 1), we first investigated whether any age-related differences in miRNA expression were observed in the three patient groups (Fig. 2). There were no age-related differences observed in either let-7c, miR-16, miR-103b or miR-106b in urine or blood samples, however, urine miR-34a expression correlated strongly with age in both the normal group and the LUTD group, but not in the TCC group (Fig. 2). Correlation between age and miR-34a was not observed in blood samples. Neither gender nor body weight correlated with the expression of any of the miRNA tested (data not shown). It is possible that suppression of miR-34a expression may occur in aging TCC patients. Age-matched control studies will be necessary to confirm this.

### Paired analysis of miRNA expression in patient urine samples demonstrate that miR-34a correlations with other miRNAs are disrupted in TCC

**Fig. 3:**
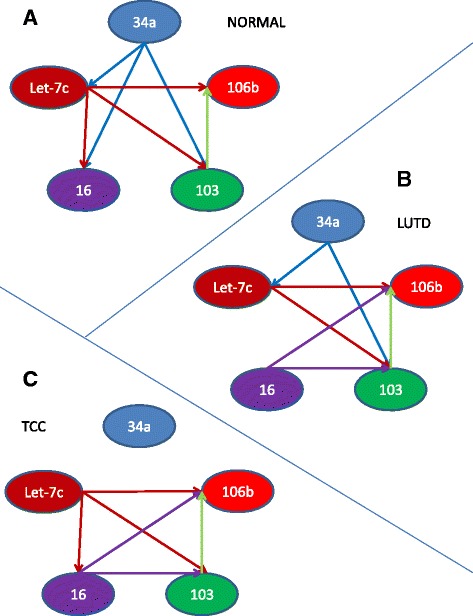
The correlation of miR-34a expression with other miRNAs is disrupted in TCC. In urine samples from normal and LUTD patients, expression of miR-34a trended with other related miRNA: let-7c, miR-16, and miR-103b, but not miR-106b (**a**). MiR-34a expression was independent of miR-16 in LUTD patients (**b**), and completely independent of any other miRNA tested in TCC (**c**). Thus, miR-34a correlation with the related miRNAs examined decreased normal > LUTD > TCC. In blood specimens from normal, LUTD, and TCC patients, expression levels of all 5 miRNA analyzed trended together in individual patients (data not shown)

As several of the miRNAs analyzed in this study target the same molecules and/or same signaling pathways (miR-34a targets; Bcl-2, CCND1, CDK4/6, CREB, DLL1, E2F3, MET, c-MYC, SIRT-1, HMGA2, Notch1, Let-7c targets; LIN28, Ras, HMGA2, c-myc, Bcl-xL, miR-16 targets; BCL2, MCL1, CCND1, WNT3A; miR-103b targets; CCNE1, CDK2, CREB1, miR-106b targets; CCND1, E2F3, RBL1/2, WEE1, [24–30]), we rationalized that there may be a correlation between their relative expression levels in individual patient specimens. We used Pearson Product Moment Correlation to assess the correlation between individual miRNA in urine and blood (Additional file 1: Tables S1–S6). In blood specimens from normal, LUTD, and TCC patients, expression levels of all 5 miRNA analyzed trended together in individual patients. In urine samples from normal patients, expression of miR-34a trended with let-7c, miR-16, and miR-103b, but not miR-106b (Fig. 3A). In urine samples from LUTD patients, expression of miR-34a trended with let-7c and miR-103b but not miR-16 or miR-106b (Fig. 3B). In urine from TCC patients, miR-34a expression was completely independent of let-7c, miR-16, miR-103b, and miR-106b (Fig. 3C). Thus, miR-34a correlation with the other miRNAs examined decreased from normal > LUTD > TCC. Conversely, miR-16 was *not* correlated with miR-103b or miR-106b in urine from normal canine but significant correlation was observed in the diseased states. These data indicate that alteration of coordinated expression of miRNAs occurs in patients with LUTD and TCC. It is noteworthy that no correlation between miRNA expression and RNA yield was observed in any of these settings.

### MiRNA expression levels in normal, LUTD, and TCC blood and urine samples

**Fig. 4:**
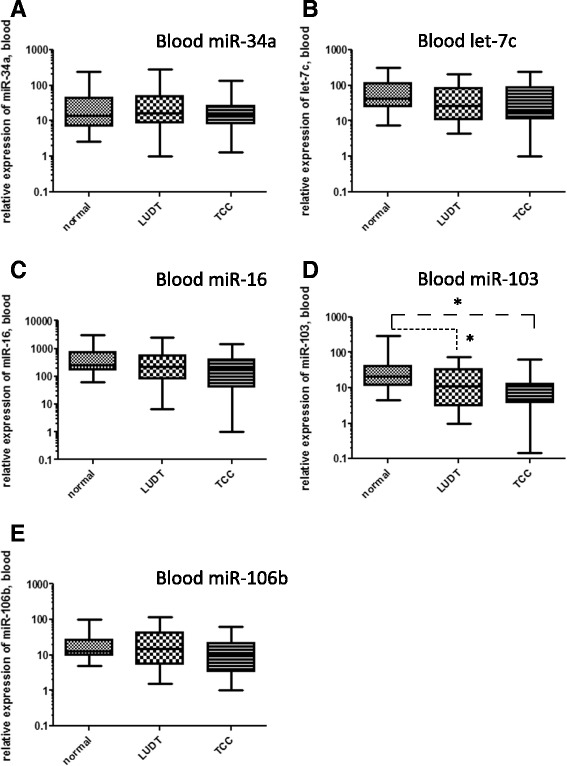
Comparison of expression levels of miR-34a, let-7c, miR-16, miR-103b, and miR-106b in blood specimens from patients with normal bladders, lower urinary tract disease (LUTD), and transitional cell carcinoma (TCC) of the bladder. One-way ANOVA on ranks identified statistically significant differences in the expression levels of miR-103b in the blood of normal vs LUTD (*p* = 0.028) and normal vs TCC patients (*p* = 0.011), but not LUTD vs TCC patients (*p* > 0.05) (**d**). No statistically significant differences in expression levels were observed between the 3 patient groups for miR-34a, let-7c, miR-16, or miR-106b (**a**, **b**, **c**, and **e**)

Next, we investigated whether any of the miRNA tested were differentially expressed in blood and urine samples from normal dogs and those with LUTD or TCC. Kruskal-Wallis one-way ANOVA on ranks (ranked ANOVA), an exploratory non-parametric method that is frequently used in biomarker discovery studies [51–56], was used for these analyses. No significant differences in expression of miR-34, let-7c, miR-16, or miR-106b were observed in blood samples from the three groups (Fig. 4A-C, E, Table 2), however, one-way ANOVA on ranks identified statistically significant differences in the expression levels of miR-103b in the blood of normal vs LUTD (*p* = 0.028) and normal vs TCC patients (*p* = 0.011), but not LUTD vs TCC patients (*p* > 0.05) (Fig. 4D, Table 2).

**Table 2:** Median values of miRNA from blood and urine of dogs with normal bladders, LUTD and TCC and one-way ANOVA on ranks analysis

Age	MEDIAN VALUES			*p*-values (one-way ANOVA on ranks)		
	NORMAL	LUTD	TCC	NORMAL vs LUTD	NORMAL vs TCC	LUTD vs TCC
	5	7	11	0.0371	<0.001	0.002
Blood miR-34a	13.882	16.393	15.047	>0.05	>0.05	>0.05
Blood Let-7c	41.478	26.022	19.420	>0.05	>0.05	>0.05
Blood miR-16	247.180	223.006	211.826	>0.05	>0.05	>0.05
Blood miR-103b	21.427	11.097	11.757	0.028	0.011	0.709
Blood miR-106b	12.663	14.910	10.452	>0.05	>0.05	>0.05
Urine miR-34a	26.152	19.947	16.305	>0.05	>0.05	>0.05
Urine Let-7c	36.801	5.603	1.788	<0.001	<0.001	0.092
Urine miR-16	37.730	64.643	21.398	0.066	0.095	0.016
Urine miR-103b	8.539	5.199	2.038	0.298	<0.001	0.002
Urine miR-106b	8.380	6.623	2.923	0.276	0.001	0.063

**Fig. 5:**
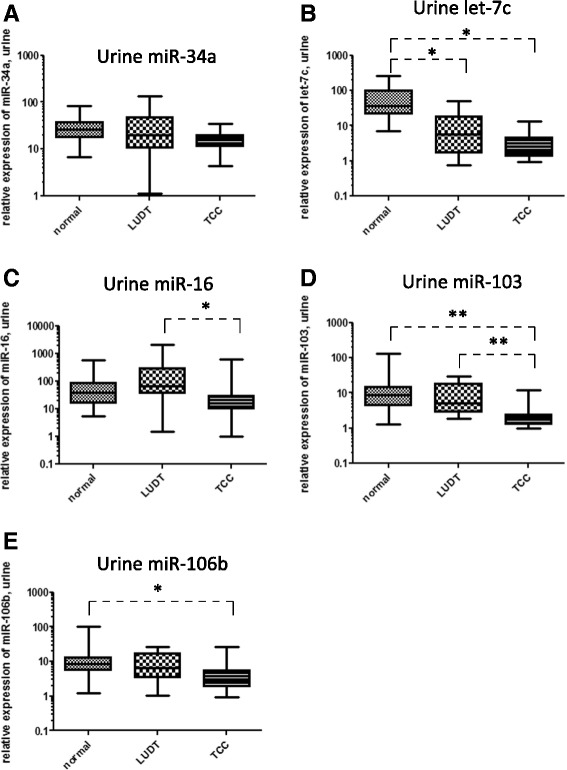
Comparison of expression levels of miR-34a, let-7c, miR-16, miR-103b, and miR-106b in urine specimens from patients with normal bladders, lower urinary tract disease (LUTD), and transitional cell carcinoma (TCC) of the bladder. One-way ANOVA on ranks identified significant differences in the expression levels of miR-16 and miR-103b in the urine of LUTD vs TCC patients (**c** and **d**, *p* < 0.05 and *p* < 0.005, respectively), of let-7c levels in the urine of normal vs TCC and LUTD patients (**b**, p < 0.05 for both), and of miR-106b levels in the urine of normal vs TCC patients (**e**, p < 0.05). A statistically significant difference in miR-34a expression levels was not observed between the 3 patient groups (**a**)

We also examined the levels of miRNA in urine from these three groups of patients (Fig. 5, Table 2). As in blood samples, there was no significant difference in miR-34a levels in the urine of animals from the three groups (Fig. 5A). A statistically significant difference in miR-106b levels in the urine of normal vs TCC patients (Fig. 2E, p<0.001) was observed. Comparison of patients with LUTD and TCC determined that only miRNA-16 (*p* = 0.016) and miR-103b (*p* = 0.002) appeared to have any significant differences (Fig. 5C, D, Table 2). Thus, these two miRNAs are potential candidates for distinguishing biomarkers of TCC vs LUTD.

## Discussion

There is a clinical need to develop non-invasive and inexpensive tests that have fast turnaround to distinguish between canine patients with non-neoplastic LUTD and canine patients with TCC. The goal of this study was to determine whether assessment of miRNA expression in canine urine and blood samples is possible and could help address this need. We successfully assessed expression of five miRNA (miR-34a, let-7c, miR-16, miR-103b, and miR-106b) in canine urine and blood samples. Analysis of miRNA in canine urine has not previously been reported. Statistically significant differences in miR-103b (p = 0.002) and miR-16 (p = 0.016) expression levels were observed in canine urine specimens from LUTD versus TCC patients, however, no difference in miRNA expression levels was observed in canine blood specimens from these 2 groups. It should be noted that this study was exploratory in nature and a larger prospective study will be necessary to validate the association of miR-103b and miR-16 expression levels with canine TCC. It should also be noted that while it has been shown that some dog breeds are predisposed to TCC [3] this study was not powered to detect whether miRNA expression contributes to these predispositions and there were too few of any one breed in the TCC and control groups to attempt meaningful analysis. Lastly, the study was not powered to determine whether these miRNA are expressed early in the course of disease or only after they progress to become muscle invasive. More work is need to determine if these miRNA are associated with earlier superficial forms of the disease.

To our knowledge, this is the first study to observe differential expression of miR-103b and miR-16 in body fluids from canine bladder cancer patients versus patients with LUTD. Jiang et al. very recently reported lower miR-103b expression levels predict worse outcome for humans with muscle invasive bladder cancer [61]. This group assessed miR-103b expression in human blood but not urine samples. It is possible lack of statistical power is a reason why we did not observe differences of miR-103b in canine blood samples in addition to urine samples. In addition, the genetics of the human bladder may be different from that of canine bladder. MiR-103b has been shown to target several molecules which play a role in carcinogenesis in other cell types, for example CCNE1, CDK2, CREB1, DICER, and PTEN [62] [44], and colorectal cancer cell line studies indicate dysregulation of miR-103b expression can drive cancer progression [62–64]. In addition to validating miR-103b as a non-invasive biomarker to distinguish between canine LUTD and TCC, our future studies will focus on identifying downstream targets of miR-103b in bladder cancer cells and the impact of miR-103b on bladder cancer cell growth and survival; it is possible that miR-103b may have utility as a therapeutic target as well as a diagnostic biomarker. The targets of miR-16 in bladder cancer cells have not been determined and its impact on bladder cancer cell growth and survival remains unknown, however, studies in other cancer cell types, including chronic lymphocytic lymphoma (CLL) and prostate cancer, have demonstrated dysregulation of miR-16 expression is associated with cancer initiation and/or progression [65–67]. The loss of correlation of miR-34a expression with age in TCC patients as well as the loss of coordinated expression of miR-34a with let-7c, miR-103b, and miR-106b (a progressive loss of miR-34a coordination with these miRNA was observed from normal > LUTD > TCC patients), indicates miR-34a may also contribute to bladder carcinogenesis. Coordinated control of miRNA expression has been shown to be important in many biological systems to allow for the regulation of complex cellular processes and can occur through genomic clustering, epigenetic regulation, or regulation by a shared transcription factor [68, 69]. In vitro studies will be necessary to determine how dysregulation of coordinated miRNA expression occurs in bladder cancer cells and how this contributes to carcinogenesis.

Key differences were observed between our current study and a prior study in which we assessed expression of miR-106b, miR-34a, miR-16, and miR-103b, and let-7c in archival paraffin-embedded tissue samples which were collected at time of necropsy or biopsy from canine patients with LUTD versus TCC [38]. In archival tissue samples, expression of miR-106b, miR-34a, miR-16, and miR-103b was higher in canine LUTD versus TCC patients. In our current study, no difference in expression of miR-106b and miR-34a was observed in blood or urine samples and miR-103b and miR-16 expression levels were decreased in urine samples from LUTD versus TCC patients. Greater variability in miRNA levels in body fluids versus tissue may account for why differences in miR-34a and miR-106b expression levels were not observed in the current study; within group variances were much higher for these miRNA in the urine and blood analyses described in the current manuscript compared to our previous archival tissue analyses. In case of miR-103b and miR-16, it is possible that the miRNA is produced in the LUTD affected tissue and is retained there, while in TCC, although produced in the tissue, they are then released into the circulation easily. In support of this, several groups have recently shown that some cancer cells can selectively export miRNA [70–72]. Hence selective export of miRNA by in TCC but not LUTD could explain why the levels of these miRNA are lower in the TCC tissue compared to LUTD, but higher in the released urine. Other studies have shown miRNA expression levels can be extremely variable in body fluid specimens [47, 48]. It is possible the difference in direction in trend of miR-16 and miR-103b in urine versus tissue samples is due, at least in part, to differences in the type of cells present in tissue versus urine samples and their relative proportion. For example, presence and/or proportion of immune cells in the urine samples from the three groups could be a factor. In the current study, 3 out of the 17 (17.6%) patients with TCC had urinary tract infections at the time of diagnosis. This rate is comparable to a recent study where 25% of dogs diagnoses with TCC had a positive urine culture prior to beginning chemotherapy [73]. It is likely the archival tissue samples contained a higher proportion of bladder cells compared to the blood and urine samples. Differences in collection and processing that urine and blood samples versus tissue samples went through may have also contributed to the observed differences. While a direct comparison of miRNA expression between matched tissue, blood, and urine samples is certainly warranted to address the observed discrepancies between the two studies it will likely prove challenging both financially and logistically as bladder biopsies are not routinely performed for canine patients and owners may not be willing to give consent for this procedure due to associated risks.

To our knowledge, extraction and assessment of miRNA from canine urine has not previously been performed, and only a limited number of studies have assessed miRNA expression using human urine samples [30–32]. Urine analysis is ideally suited to biomarker discovery studies for TCC and other urological diseases because urine is in direct contact with the bladder and it can easily be obtained from patients. The fact that we observed statistically significant differences in levels of 4 of the 5 miRNAs assessed in urine specimens (differential expression of miR-103b and miR-16 in LUTD versus TCC patients, and differential expression of let-7c and miR-106b in clinically normal versus LUTD and/or TCC patients) versus only 1 in blood specimens (differential expression of miR-103b in clinically normal versus TCC patients) between the 3 patient groups, and also observed progressive loss of coordinated expression of miR-34a, supports the use of urine specimens rather than blood specimens for urological biomarker discovery studies.

## Conclusions

In summary, our data demonstrate an association exists between miR-103b and miR-16 expression levels in urine and TCC, and show that miRNA can be isolated and quantified in canine urine as well as blood specimens. Our results indicate that further investigation of these miRNA as diagnostic non-invasive biomarkers for canine TCC is warranted.

## Additional files

Additional file 1: Table S1. *P* values for correlation of miRNA expression in RNA extracted from blood samples from canine patients with normal bladders, **Table S2.** *P* values for correlation of miRNA expression in RNA extracted from urine samples from canine patients with normal bladders, **Table S3.** P values for correlation of miRNA expression in RNA extracted from blood samples from canine patients with LUTD, **Table S4.** *P* values for correlation of miRNA expression in RNA extracted from urine samples from canine patients with LUTD, **Table S5.** *P* values for correlation miRNA expression in RNA extracted from blood samples from canine patients with TCC, **Table S6.** *P* values for correlation of miRNA expression in RNA extracted from urine samples from canine patients with TCC. (DOCX 28 kb)

Additional file 2: Table S7. Demographic and miRNA expression data. (XLSX 25 kb)

## Abbreviations

AUC: Area under the curve
IACUC: Institutional animal care and use committee
LUTD: Lower urinary tract disease
miRNA: MicroRNA
OR: Odds ratio
ROC: Receiver operating characteristic
SD: Standard deviation
TCC: Transitional cell carcinoma
TNM: Tumor, Lymph Node and Metastasis
UTI: Urinary tract infection
