# Supplementary material for: MicroRNA profiling of dogs with transitional cell carcinoma of the bladder using blood and urine samples
Source: BMC Vet Res. 2017 Nov 15;13:339. doi: 10.1186/s12917-017-1259-1 (PMC5688639; doi:10.1186/s12917-017-1259-1)
Supplement: Supplementary file 1 — P values for correlation of miRNA expression in RNA extracted from blood samples from canine patients with normal bladders, Table S2. P values for correlation of miRNA expression in RNA extracted from urine samples from canine patients with normal bladders, Table S3. P values for correlation of miRNA expression in RNA extracted from blood samples from canine patients with LUTD, Table S4. P values for correlation of miRNA expression in RNA extracted from urine samples from canine patients with LUTD, Table S5. P values for correlation miRNA expression in RNA extracted from blood samples from canine patients with TCC, Table S6. P values for correlation of miRNA expression in RNA extracted from urine samples from canine patients with TCC. (DOCX 28 kb) [file 12917_2017_1259_MOESM1_ESM.docx]

**Table S1:** P values for correlation of miRNA expression in RNA extracted from blood samples from canine patients with normal bladders

| **BLOOD** | let-7c | miR-16 | miR-103 | miR-106b | ng/ul RNA | ng RNA |
| --- | --- | --- | --- | --- | --- | --- |
| miR-34a | 0.884 | 0.881 | 0.921 | 0.928 | 0.0459 | 0.0459 |
|  | p<0.001 | p<0.001 | p<0.001 | p<0.001 | 0.817 | 0.817 |
|  | 28 | 28 | 28 | 28 | 28 | 28 |
|  |  |  |  |  |  |  |
| Let-7c |  | 0.953 | 0.938 | 0.933 | -0.0654 | -0.0654 |
|  |  | p<0.001 | p<0.001 | p<0.001 | 0.741 | 0.741 |
|  |  | 28 | 28 | 28 | 28 | 28 |
|  |  |  |  |  |  |  |
| miR-16 |  |  | 0.952 | 0.927 | -0.0283 | -0.0283 |
|  |  |  | p<0.001 | p<0.001 | 0.886 | 0.886 |
|  |  |  | 28 | 28 | 28 | 28 |
|  |  |  |  |  |  |  |
| miR-103 |  |  |  | 0.948 | 0.0456 | 0.0456 |
|  |  |  |  | p<0.001 | 0.818 | 0.818 |
|  |  |  |  | 28 | 28 | 28 |
|  |  |  |  |  |  |  |
| miR-106b |  |  |  |  | 0.0705 | 0.0705 |
|  |  |  |  |  | 0.722 | 0.722 |
|  |  |  |  |  | 28 | 28 |
|  |  |  |  |  |  |  |
| Ng/ul RNA |  |  |  |  |  | 1.000 |
|  |  |  |  |  |  | 9.620E-203 |
|  |  |  |  |  |  | 28 |

**Table S2:** P values for correlation of miRNA expression in RNA extracted from urine samples from canine patients with normal bladders

| **URINE** | let-7c | miR-16 | miR-103 | miR-106b | ng/ul RNA | ng RNA |
| --- | --- | --- | --- | --- | --- | --- |
| miR-34a | 0.510 | 0.666 | 0.458 | 0.371 | -0.154 | -0.154 |
|  | 0.0056 | 0.00011 | 0.0144 | 0.0518 | 0.433 | 0.433 |
|  | 28 | 28 | 28 | 28 | 28 | 28 |
|  |  |  |  |  |  |  |
| Let-7c |  | 0.450 | 0.501 | 0.406 | -0.167 | -0.167 |
|  |  | 0.0162 | 0.00663 | 0.0321 | 0.396 | 0.396 |
|  |  | 28 | 28 | 28 | 28 | 28 |
|  |  |  |  |  |  |  |
| miR-16 |  |  | 0.205 | 0.109 | -0.160 | -0.160 |
|  |  |  | 0.296 | 0.583 | 0.416 | 0.416 |
|  |  |  | 28 | 28 | 28 | 28 |
|  |  |  |  |  |  |  |
| miR-103 |  |  |  | 0.969 | 0.138 | 0.138 |
|  |  |  |  | <0.001 | 0.484 | 0.484 |
|  |  |  |  | 28 | 28 | 28 |
|  |  |  |  |  |  |  |
| miR-106b |  |  |  |  | 0.275 | 0.275 |
|  |  |  |  |  | 0.156 | 0.156 |
|  |  |  |  |  | 28 | 28 |
|  |  |  |  |  |  |  |
| Ng/ml RNA |  |  |  |  |  | 1.000 |
|  |  |  |  |  |  | 0.000 |
|  |  |  |  |  |  | 28 |

**Table S3:** P values for correlation of miRNA expression in RNA extracted from blood samples from canine patients with LUTD

| **BLOOD** | let-7c | miR-16 | miR-103 | miR-106b | ng/ul RNA | ng RNA |
| --- | --- | --- | --- | --- | --- | --- |
| miR-34a | 0.751 | 0.449 | 0.703 | 0.649 | -0.132 | -0.132 |
|  | 0.000024 | 0.028 | ,,<0.001 | 0.0006 | 0.539 | 0.539 |
|  | 24 | 24 | 24 | 24 | 24 | 24 |
|  |  |  |  |  |  |  |
| Let-7c |  | 0.639 | 0.766 | 0.730 | 0.0326 | 0.0326 |
|  |  | ,,<0.001 | ,,<0.001 | ,,<0.001 | 0.880 | 0.880 |
|  |  | 24 | 24 | 24 | 24 | 24 |
|  |  |  |  |  |  |  |
| miR-16 |  |  | 0.797 | 0.838 | -0.0797 | -0.0797 |
|  |  |  | ,,<0.001 | ,,<0.001 | 0.711 | 0.711 |
|  |  |  | 24 | 24 | 24 | 24 |
|  |  |  |  |  |  |  |
| miR-103 |  |  |  | 0.884 | 0.0654 | 0.0654 |
|  |  |  |  | ,,<0.001 | 0.761 | 0.761 |
|  |  |  |  | 24 | 24 | 24 |
|  |  |  |  |  |  |  |
| miR-106b |  |  |  |  | -0.0134 | -0.0134 |
|  |  |  |  |  | 0.950 | 0.950 |
|  |  |  |  |  | 24 | 24 |
|  |  |  |  |  |  |  |
| Ng/ul RNA |  |  |  |  |  | 1.000 |
|  |  |  |  |  |  | 9.411E-172 |
|  |  |  |  |  |  | 24 |

**Table S4:** P values for correlation of miRNA expression in RNA extracted from urine samples from canine patients with LUTD

| **URINE** | let-7c | miR-16 | miR-103 | miR-106b | ng/ul RNA | ng RNA |
| --- | --- | --- | --- | --- | --- | --- |
| miR-34a | 0.852 | 0.306 | 0.695 | 0.450 | -0.197 | -0.197 |
|  | ,,<0.001 | 0.217 | 0.0014 | 0.0609 | 0.419 | 0.419 |
|  | 19 | 18 | 18 | 18 | 19 | 19 |
|  |  |  |  |  |  |  |
| Let-7c |  | 0.250 | 0.626 | 0.567 | -0.219 | -0.219 |
|  |  | 0.317 | 0.0055 | 0.0142 | 0.367 | 0.367 |
|  |  | 18 | 18 | 18 | 19 | 19 |
|  |  |  |  |  |  |  |
| miR-16 |  |  | 0.837 | 0.575 | -0.0218 | -0.0218 |
|  |  |  | ,,<0.001 | 0.0125 | 0.932 | 0.932 |
|  |  |  | 18 | 18 | 18 | 18 |
|  |  |  |  |  |  |  |
| miR-103 |  |  |  | 0.663 | -0.0810 | -0.0810 |
|  |  |  |  | 0.0027 | 0.749 | 0.749 |
|  |  |  |  | 18 | 18 | 18 |
|  |  |  |  |  |  |  |
| miR-106b |  |  |  |  | 0.143 | 0.143 |
|  |  |  |  |  | 0.570 | 0.570 |
|  |  |  |  |  | 18 | 18 |
|  |  |  |  |  |  |  |
| Ng/ul RNA |  |  |  |  |  | 1.000 |
|  |  |  |  |  |  | 0.000 |
|  |  |  |  |  |  | 20 |

**Table S5:** P values for correlation miRNA expression in RNA extracted from blood samples from canine patients with TCC

| **BLOOD** | let-7c | miR-16 | miR-103 | miR-106b | ng/ul RNA | ng RNA |
| --- | --- | --- | --- | --- | --- | --- |
| miR-34a | 0.634 | 0.800 | 0.908 | 0.922 | -0.0423 | -0.0423 |
|  | 0.00628 | 0.000117 | ,,<0.001 | ,,<0.001 | 0.872 | 0.872 |
|  | 17 | 17 | 16 | 17 | 17 | 17 |
|  |  |  |  |  |  |  |
| Let-7c |  | 0.689 | 0.790 | 0.523 | -0.187 | -0.187 |
|  |  | 0.00220 | ,,<0.001 | 0.0312 | 0.473 | 0.473 |
|  |  | 17 | 16 | 17 | 17 | 17 |
|  |  |  |  |  |  |  |
| miR-16 |  |  | 0.931 | 0.888 | -0.0123 | -0.0123 |
|  |  |  | ,,<0.001 | ,,<0.001 | 0.962 | 0.962 |
|  |  |  | 16 | 17 | 17 | 17 |
|  |  |  |  |  |  |  |
| miR-103 |  |  |  | 0.892 | -0.0338 | -0.0338 |
|  |  |  |  | ,,<0.001 | 0.901 | 0.901 |
|  |  |  |  | 16 | 16 | 16 |
|  |  |  |  |  |  |  |
| miR-106b |  |  |  |  | -0.00421 | -0.00421 |
|  |  |  |  |  | 0.987 | 0.987 |
|  |  |  |  |  | 17 | 17 |
|  |  |  |  |  |  |  |
| Ng/ul RNA |  |  |  |  |  | 1.000 |
|  |  |  |  |  |  | 0.000 |
|  |  |  |  |  |  | 17 |

**Table S6:** P values for correlation of miRNA expression in RNA extracted from urine samples from canine patients with TCC

| **URINE** | let-7c | miR-16 | miR-103 | miR-106b | ng/ul RNA | ng RNA |
| --- | --- | --- | --- | --- | --- | --- |
| miR-34a | 0.272 | 0.191 | 0.285 | 0.293 | -0.0740 | -0.0740 |
|  | 0.418 | 0.574 | 0.395 | 0.381 | 0.829 | 0.829 |
|  | 11 | 11 | 11 | 11 | 11 | 11 |
|  |  |  |  |  |  |  |
| Let-7c |  | 0.761 | 0.908 | 0.735 | -0.198 | -0.198 |
|  |  | 0.00653 | 0.00011 | 0.00998 | 0.559 | 0.559 |
|  |  | 11 | 11 | 11 | 11 | 11 |
|  |  |  |  |  |  |  |
| miR-16 |  |  | 0.953 | 0.985 | 0.00437 | 0.00437 |
|  |  |  | ,,<0.001 | ,,<0.001 | 0.990 | 0.990 |
|  |  |  | 11 | 11 | 11 | 11 |
|  |  |  |  |  |  |  |
| miR-103 |  |  |  | 0.940 | -0.0562 | -0.0562 |
|  |  |  |  | ,,<0.001 | 0.870 | 0.870 |
|  |  |  |  | 11 | 11 | 11 |
|  |  |  |  |  |  |  |
| miR-106b |  |  |  |  | 0.0910 | 0.0910 |
|  |  |  |  |  | 0.790 | 0.790 |
|  |  |  |  |  | 11 | 11 |
|  |  |  |  |  |  |  |
| Ng/ul RNA |  |  |  |  |  | 1.000 |
|  |  |  |  |  |  | 9.370E-072 |
|  |  |  |  |  |  | 11 |
